# Perceptions and Experiences About Device-Emitted Radiofrequency Radiation and Its Effects on Selected Brain Health Parameters in Southwest Nigeria

**DOI:** 10.7759/cureus.18211

**Published:** 2021-09-23

**Authors:** Joshua Owolabi, Olayinka S Ilesanmi, Amitabye Luximon-Ramma

**Affiliations:** 1 Anatomy/Neuroscience, Babcock University, Ilishan-Remo, NGA; 2 Anatomy/Neuroscience, University of Global Health Equity, Kigali, RWA; 3 Community Medicine, University of Ibadan, Ibadan, NGA; 4 Health Sciences, University of Technology, Port Louis, MUS

**Keywords:** mental health, sleep disorder, headache, fatigue, perception, radiofrequency radiation

## Abstract

Introduction

Radiofrequency radiation (RFR) is a form of non-ionising radiation that is used or emitted by a number of technologies and innovative devices including mobile phones and computers and gadgets. Exposure to RFR has been reported to have certain negative effects on human health. It is clear that quality and reliable data will be required with respect to the specific nature of RFR effects on mental health. This research considered the perceptions and exposure-related experiences of people within a Nigerian population with respect to RFR.

Methods

Structured and validated questionnaires were used to profile self-reported patterns of behaviour and sleep in humans. Questionnaire administration-electronic was opened for exactly one week, consisting of 25 specific questions and five open-ended questions [total = 30 questions]. A total population approach was adopted [N=~240]. Bivariate analysis using Chi-square tests were conducted to determine the association between knowledge of electronic gadgets as a source of radiofrequency radiation and sociodemographic characteristics of respondents. Binary logistic regression was used to determine the factors associated with good knowledge of electronic gadgets as a source of radiofrequency radiation. The level of statistical significance was set at p ≤ 0.05.

Results

The response rate was approximately 84%. Fatigue/tiredness [69.6%], attention deficit [69.1%] and headache [62.4%] ranked top amongst RFR-associated negative effects on mental health. Among the respondents, 29 (56.9%) among those above 20 years had good knowledge of radiofrequency radiation from electronic gadgets compared to 72 (47.2%) aged 20 years and below (X^2^ = 1.285, p = 0.257). Also, 45 (59.2%) of persons who lived in a town/village had good knowledge of radiofrequency radiation from electronic gadgets compared to 56 (44.4%) who lived in the city (X^2^ = 4.135, p = 0.042). Persons who lived in a town/village had nearly two times the odds of having good knowledge of RFR from electronic gadgets.

Conclusion

The study showed that respondents had experienced significant and negative effects of RFR on their mental health. The current level of knowledge and awareness on the nature of RFR and exposures was just about average, indicating a critical and urgent need to educate the public on the subject.

## Introduction

Radio-frequency radiation (RFR) belongs to the electromagnetic wave spectrum in the frequency of the waves range between ~500 kilohertz - 2,000 megahertz. RFR enables several devices to function through wave transmissions such as in the case of Wi-Fi-enabled phones and computer devices. Humans on the planet earth are exposed to certain natural RFR radiations which mainly include the sun, the atmosphere such as during lightning, and the earth natural electromagnetic field. Major artificial sources of RFR include the broadcasting radio and television signals, wireless phones transmitting signals - phones, cell phone towers, satellite sources etc., radar, Wi-Fi devices, Bluetooth® devices, and smart meters and scanners, e.g., millimetre-wave scanners such as full-body scanners for security screening. To put things in perspective, electromagnetic field radiations are tagged radiofrequency radiation when the frequencies of the waves range between ~500 kilohertz (500 kHz = 500,000 waves per second) to 2,000 megahertz (2,000 MHz = two billion waves per second) [1]. 

Potential negative effects of radiations in the form of radiofrequency have been reported, including an increased risk of neurodegenerative diseases. Also, alarm has been raised in different quarters on the potential negative effects such might have on brain development and mental functions which altogether could have significant effects on mental health. RFR has been reported to affect auditory mechanisms in experimental models [2]; cognition and associated brain attributes in teenagers [3]. It has also been linked to other brain health aberrations, including epilepsy [4]. Much is obviously yet to be known about the mechanisms of such effects and the extent of the suspected negative effects. On the other hand, a number of counterclaims have been made to allay fears and state that the level and amount of exposure that comes from the basic or routine daily use of radiofrequency in phones and other wireless devices might not be harmful to the brain. For example, experimental exposure of cultured cells to radiofrequency radiations caused whole cells morphological aberrations, cellular DNA damage, cell cycle arrest, oxidative stress and reactive oxygen species formation [5].

Relatively long before now, there were indications that RFR might influence neural activities, hence causing neurological disturbances [6, 7]. This was considered to be an indication of what other effects RFR might have on body functions. While there are several discrete reports on effects and potential risks, Singh and Kapoor [8], would rather state their positions that these data would not provide conclusive evidence on exact effects but would rather recommend quality precautionary measures. The implications of this position would be that there is always evidence that suggests risks and there is a need to carefully and objectively consider them through thorough research and careful evidence extrapolations. 

Sleep is one of the behavioural parameters that has been studied relatively extensively in terms of how RFR might affect its quality in studies of humans. Arousal has also been studied in association with RFR exposure effects on sleep, and isolation relative to sleep. It has been shown that RFR effects could influence the quality of sleep in individuals [9], causing a reduction in arousal [10], having effects on the latency of sleep [11], and causing measurable sleep disturbance and dysfunctions [during the day] especially in females [12].

Other studies have looked into the effects of RFR on certain other behavioural parameters which include learning, cognition, memory and attention among others. Thomas et al. [13] had reported that RFR caused behavioural problems in adolescents. While the submission might appear relatively vague, Zheng et al. [14] had reported that such effects specifically included inattention in adolescents, as a result of their use of cell phones. Cognitive functions have also been explored in terms of our RFR and might impair or affect it specifically or influence its associated parameters. Quality evidence exists from literature that RFR could impact the quality of cognitive functions in humans even with exposures that lasted for only minutes [15, 16]. Animal studies showed that such effects might include impairment of cognitive functions [17]. RFR effects have also been linked to hyperactivity in animals or what was described as hyperactivity-like behaviour [18,19].

Lai [20] emphasised that most animal studies showed that RFR had effects on behavioural parameters, however, more human studies had reported that RFR had no such effects. The author had attributed such variations to either the variations in the biological milieu of human versus experimental animals or the variations in the experimental regimens of exposure versus the human real exposure patterns. There is merit in such arguments. However, it should be noted that modelled experimentation has remained inseparable and indispensable to biomedical sciences and as such, in the past have given highly reliable data. What one might advocate for going forward is the need to carefully and accurately model experimental studies after patterns of human exposure and to carefully measure the effects in manners that could provide accurate extrapolations. One thing that might not be divorceable from the crisis of lack of consensus in the nature of RFR is the significant political and economical vested interests that often show in how stakeholders in the world of business and in government often choose to select what might constitute their body of evidence.

The primary objective of this study was to study the knowledge, awareness, and perceptions as well as exposure-related experiences of people within a Nigerian population with respect to RFR exposure.

## Materials and methods

Study area population

The questionnaire was administered to faculty members, staff and students of the Department of Anatomy at the Ben Carson School of Medicine, Babcock University, in Ogun State, Nigeria. This consisted of the Nigerian population whose understanding of RFR at the basic level qualified them to be relatively knowledgeable to provide informed responses to the questions as contained in the questionnaire. This would be needful to provide some generalizability to the research findings. Also, the use of RFR and neighbours’ devices was an essential component of daily activities sessions delivered to students in the last one year have been required to actively learn through their computer devices including laptops desktops and other handheld devices. Some remote lecture sessions have been held using computer devices.

The primary domain that these people were or lived in was the University. The University had internet services provided for approximately 24 hours daily through servers and routers to connect computer devices especially laptops, mobile phones, including other handheld devices such as tablets and iPad among others. Also, on a daily basis, the secondary domain of residence was the respondents’ homes. Because they were required to connect to the internet to have access not just the learning management system but to other learners irrespective of the difference in location, it was also required that they actively used RFR-enabled devices to achieve learning and connection to the communities of learning. Quality internet penetrance was assured for approximately 24 hours on daily basis from respondents’ homes, whether sited in rural or urban locations.

Study design

Questionnaire Administration: Total Community 

The questionnaire (see Appendices), consisting of ~25 specific questions and ~5 open-ended questions [total = ~30 questions] related to the research topic collected information from respondents on specific themes that included the following:

- Demographics: This helped to obtain quality background information from respondents.

- Knowledge of RFR effects: This theme section consisted of questions that collected data about respondents’ awareness about the range of effects that are attributable to RFR exposure. 

- Exposure patterns: This helped to collect data from respondents on their habitual pattern RFR-enable technology use, the dose of exposure per and the duration of exposure.

- Experience with exposure: This section collected data about the experiences of the respondents with the use of RFR which are relevant to the subject, objectives and context of the current study.

- Perceptions: This section collected data on the perception of the respondents about RFR exposure generally, and more specifically, through the use of RFR-enabled gadgets or devices. 

- Open-ended questions: These included 3-5 statements from response on safety, exposure patterns and policy/regulation matters about RFR exposure.

Sample Size and Sampling Method

This study considered a department of the population over a total population of 240 people including faculty members, support staff members and students. The population by virtue of its natural distribution had a higher percentage of females. Students who were younger constituted the largest percentage of the population, being about 90%. Faculty and staff members made up about 10% of the target population. An electronic questionnaire, as Google forms, was delivered to each person through at least one personal platform including either or both of the individual student official email as assigned by the University or the locally used social media platform for communication, which is WhatsApp. Each targeted participant was required to indicate consent before starting the questionnaire, thus, the principle of informed consent was strictly adhered to. Questionnaire administration was opened for exactly one week and the response rate was approximately 84%, which could be considered very high for such a research context and population.

Population attributes:

- Total population ~240; females had a higher percentage

- Students = 90% of population

- Faculty = 10%

- Responses recorded = 202

- Response rate = 84%

Data Collection Instrument

Structured questionnaires were used to profile the patterns of behaviour and sleep in humans and data were analysed to observe possible links between RFR exposure and selected behavioural parameters. Information was collected about behavioural changes that are related to fatigue, anxiety and stress levels. Questionnaire administration was opened for exactly one week and the response rate was approximately 84%.

Data Analysis

Sociodemographic characteristics were presented as properly annotated figures. Other quantitative data were presented as graphs and tables. A score of “1” was assigned to each positive response provided for the following statements: “Radiofrequency is a form of non-ionizing radiation”, “Mobile phones when connected to the internet could emit radiofrequency radiation”, “Computer devices when connected to the internet emit or use radiofrequency radiation”, and “Wi-Fi routers and modems when used for internet connection use or emit radiofrequency radiation”. Using a cut-off of 50% for the aggregate score, respondents that had a score ≥3 were categorized as having “Good knowledge of electronic gadgets as a source of RFR”, while those with scores ≤2 were categorized as having “Poor knowledge of electronic gadgets as a source of RFR”. Bivariate analysis using Chi-square tests were conducted to determine the association between knowledge of electronic gadgets as a source of radiofrequency radiation and sociodemographic characteristics of respondents. Binary logistic regression was used to determine the factors associated with good knowledge of electronic gadgets as a source of radiofrequency radiation. The level of statistical significance was set at p ≤ 0.05.

Ethics

The human participants were required to complete a questionnaire and necessary ethical issues were considered as follows:

- The participants were requested to consider the written informed consent information. Their informed consent was a requirement for participation and anyone who declined was exempted without coercion or compulsion. 

- Participation was purely voluntary without any form of coercion.

- The study involved the use of a questionnaire with a set of questions that would in no way affect participants emotionally or stress them mentally in the process of completion. 

- All respondents remained anonymous; through adherence to the principle of anonymity, the confidentiality of the participants was ensured. Results were turned in without any personal information that might indicate identity. Coding was used for any other indicative information such as demographics including gender.

- The immediate community and the global communities would benefit from the findings of the research through publications and public presentation of the finalised results. Individual participants who requested the summative findings would be given the opportunity to have the finalised reports for their benefit.

The process of data collection was humane, responsible and respectful as much as possible. The efforts of participants were duly appreciated with the promise of using the outcome of research for the benefit of mankind. Ethical approval for this body of work including its experimental [modelled] component had been sought and obtained from the Babcock University Health Research Ethical Committee (BUHREC), with an ethical clearance number BUHREC NO: 814/18.

## Results

The 202 respondents who appropriately completed the questionnaire were all Nigerians who were also living in Nigeria at the time of the study. All responses that were considered had indicated their informed consent. Among them, 182 (90.0%) were members of the student population. The response rate was 84.0%. Overall, 151 (74.8%) were below 20 years and 146 (72.3%) were females (Table 1).

**Table 1:** Table showing the sociodemographic characteristics of respondents

Variables	Frequency	%
Age (Years)		
≤20	151	74.8
>20	51	25.2
Sex		
Male	56	27.7
Female	146	72.3
Location		
Town/Village	76	37.6
City	126	62.4

Among the respondents, 113 (55.9%) were knowledgeable that computer devices when connected to the internet emit or use RFR. Also, 111 (55.0%) had the knowledge that mobile phones when connected to the internet could emit RFR (Table 2).

**Table 2:** Table showing respondents’ knowledge of radiofrequency radiation from electronic gadgets as sources of electronic radiation

Variables	Frequency	%
Radiofrequency is a source of non-ionizing radiation		
Yes	96	47.5
No	106	52.5
Mobile phones when connected to the internet could emit radiofrequency radiation		
Yes	111	55.0
No	91	45.0
Computer devices when connected to the internet emit or that radiofrequency radiation		
Yes	113	55.9
No	89	44.1
Wi-Fi routers and modems when used for internet connection use or emit radiofrequency radiation		
Yes	107	53.0
No	95	47.0

On an average, 31 (16.0%) respondents spent 6 hours or less on an RFR-emitting device, 87 (45.4%) spent between 7 and 12 hours, while 73 (38.2%) spent more than 12 hours on an RFR-emitting device every day, with 166 (88.3%) using these devices every day of the week (Table 3).

**Table 3:** Table showing respondents’ general pattern of using RFR-emitting devices and type of internet-enabled device used in the past one week *Television and Bluetooth RFR: radiofrequency radiation

Variables	Frequency	%
General pattern of using RFR-emitting devices		
Mobile phone		
Yes	189	97.9
No	4	2.1
Laptop		
Yes	148	76.7
No	45	23.3
Desktop		
Yes	38	19.7
No	155	80.3
Tablet		
Yes	14	60.9
No	9	39.1
Others*		
Yes	3	12.0
No	22	88.0
Type of internet-enabled device used in the past one week		
Yes	179	88.6
No	23	11.4
Used mobile phone		
Yes	158	88.8
No	20	11.2
Used Computers		
Yes	118	66.3
No	60	33.7
Used Modem or Wi-Fi		
Yes	158	88.8
No	20	11.2
Used television		
Yes	175	98.3
No	3	1.7

**Figure 1:**
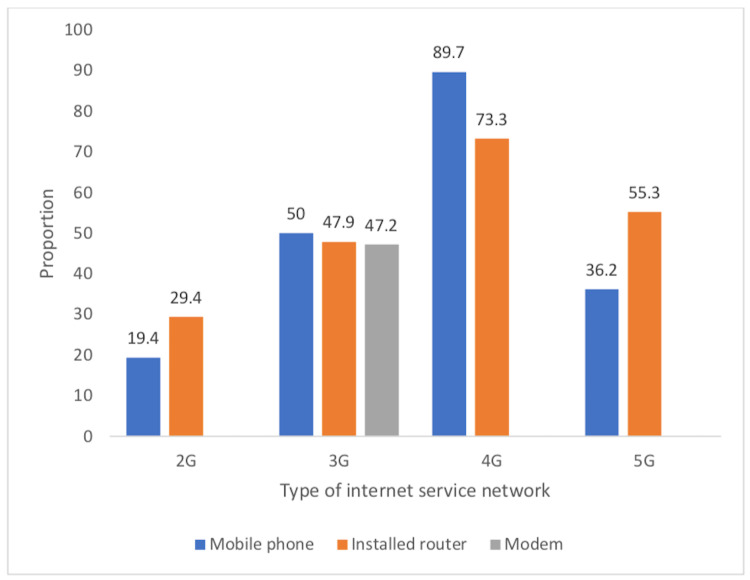
Chart showing respondents type of internet sources; data included the frequency and pattern of the use of internet service network types across different radiofrequency-emitting devices

Figure 1 shows the frequently used pattern of internet service network types among the respondents. Among mobile phone users, the frequency of use of 2G was 14 (19.4%), 3G was 58 (50.0%), 4G was 156 (89.7%), and 5G was 17 (36.2%). Among users of installed routers, 2G was used by 15 (29.4%), 3G was used by 34 (47.9%), while 4G was used by 110 (73.3%). Among modem users, 34 (47.2%) used 3G, while other internet service network types were not used (Figure 1).

**Figure 2:**
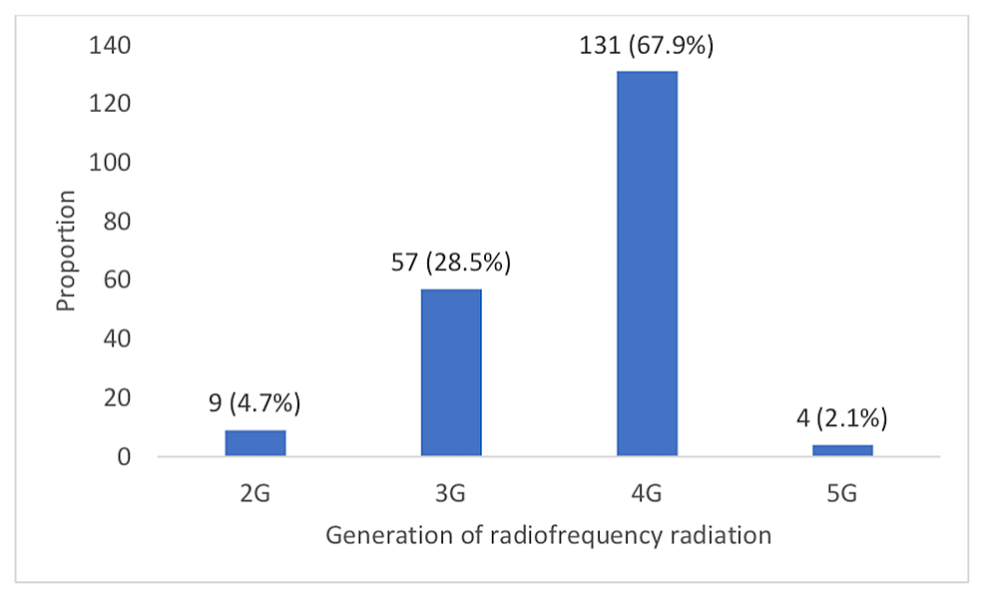
Chart showing the generation of the radiofrequency radiation emitting devices as indicated by respondents

Figure 2 presented information on the use of RFR and the generations. Regarding the routine use of generation of radiofrequency radiation, nine (4.7%) used 2G, 57 (28.5%) used 3G, 131 (67.9%) used 4G, while four (2.1%) used 5G (Figure 2).

**Figure 3:**
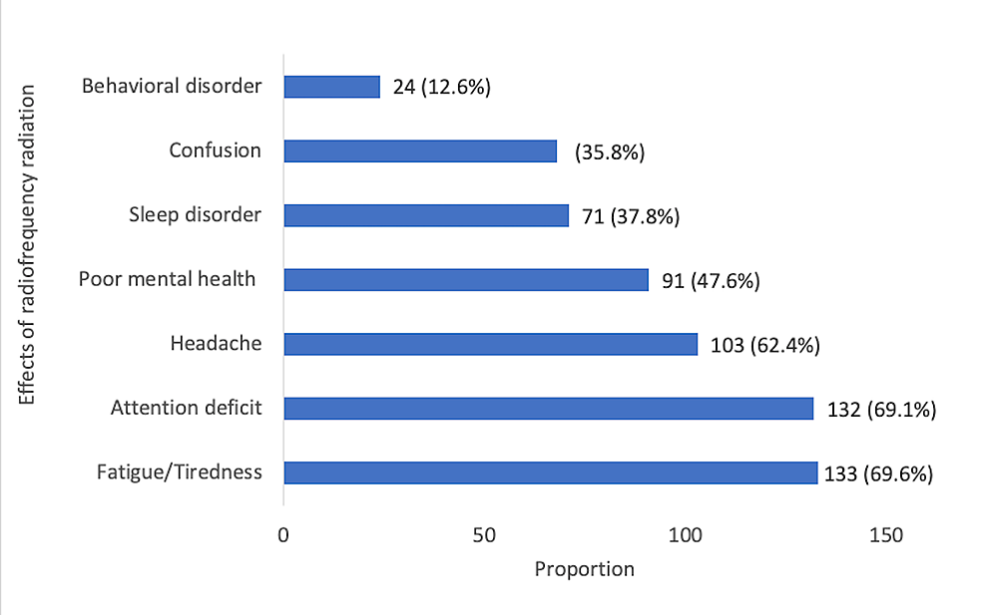
Chart showing self-reported effects of exposure to RFR-emitting devices RFR: radiofrequency radiation

Figure 3 shows the self-reported effects of exposure to RFR-emitting devices. Fatigue was reported by 133 (69.6%), attention deficit among 132 (69.1%), and headache among 103 (62.4%). Other self-reported effects of exposure to RFR-emitting devices are as shown in Figure 3.

In the last six months, 44 (27.0%) respondents reported that they had been diagnosed with mood disorders, 32 (20.5%) with anxiety disorders, 10 (7.0%) with personality disorder, 10 (7.0%) with post-traumatic stress disorder, and three (2.1%) with psychotic disorders. Overall, 101 (50.0%) had good knowledge of RFR, and 101 (50.0%) had poor knowledge of RFR. 

Among the respondents, 29 (56.9%) among those above 20 years had good knowledge of radiofrequency radiation from electronic gadgets compared to 72 (47.2%) aged 20 years and below (X2 = 1.285, p = 0.257). Also, 45 (59.2%) of persons who lived in a town/village had good knowledge of radiofrequency radiation from electronic gadgets compared to 56 (44.4%) who lived in the city (X2 = 4.135, p = 0.042). Persons who lived in a town/village had nearly two times the odds of having good knowledge of RFR from electronic gadgets (Table 4).

**Table 4:** Table showing the bivariate and multivariate analysis between knowledge of electronic gadgets as a source of radiofrequency radiation and sociodemographic characteristics of respondents *Level of significance = p < 0.05

Variables	Knowledge of radiofrequency radiation from electronic gadgets		X^2^	p-value*
	Good	Poor		
	n (%)	n (%)		
Age (Years)				
≤20	72 (47.7)	79 (52.3)	1.285	0.257
>20	29 (56.9)	22 (43.1%)		
Sex				
Male	30 (53.6)	26 (46.4)	0.395	0.530
Female	71 (48.6)	75 (51.4)		
Location				
Town/Village	45 (59.2)	31 (40.8)	4.135	0.042
City	56 (44.4)	70 (55.6)		
Determinant of good knowledge of radiofrequency radiation from electronic gadgets				
Variable	Odds Ratio	95% Confidence interval		p-value
Lives in a town/Village	1.815	1.019	3.231	0.043
Lives in a city	1			

## Discussion

To begin with, respondents in the current study clearly associated a number of mental health problems with their use of technologies that had exposed them the radiofrequency radiation. They lived in a similar geographical location and shared relevant demographic features that made them habitual users of technologies and computers (Figure 1). They also acknowledged that they were exposed to RFR as a result of using certain computers and electronic devices (Tables 2, 3). These RFR exposure-associated effects as indicated had included fatigue (69.6%), attention deficit (69.1%), headache (62.4%), poor mental health (47.6%), sleep disorder 37.8%), confusion and behavioural disorders (generally), 35.8%) (Figure 3). It is also important to know that statistical analysis of relevant parameters showed a significant relationship as such. The implication of these will be that in the studied population, these mental health-related problems could be associated with people’s exposure to radiofrequency radiation.

Several studies have reported deleterious effects of RFR with emphasis on tumorigenesis as well as on learning, memory, anxiety, and locomotion [21]. It would also appear that a larger proportion of RFR-related studies have been focused on oncology-related risks, and relatively less on mental health. This study however enriches the body of knowledge on RFR-associated risks in relation to mental health. Many respondents in this study had associated their use of RFR-enabled devices with headaches. This is in line with a report from a previous study about the link between the RFR from mobile devices and migraine headaches [22]. In fact, it was reported that headache is one of the main disorders that RFR might trigger quite often [23]. In addition to headache, another earlier study has associated RFR exposure with fatigue, and this also would align with the findings of this study [24]. Sleep disturbance is the main problem that had been reportedly associated with RFR [25]. It was further postulated that RFR could affect sleep by increasing the power of the Alpha waves during sleep [26]. The primary mechanism through which such effects were produced had been linked with aberrations in the brain dopaminergic system [27]. Also, Frey [28], had reported specifically disturbances in the brain’s dopamine-opioid system, including effects on the blood-brain barrier. Notably, the current study also showed marked elevations in the activities of dopamine in the brain at birth and at puberty, suggesting a sustained alteration in the brain dopamine system under continuous exposure to RFR.

With respect to the generation of the RFR-enabled devices that serve as internet connections, most users indicated that they used the 4G RFR devices (Figures 1, 2). This would be in line with the reality in the current instance as the country uses the 4G network as the most advanced generation, although, a few people still used the lower generations especially 3G. Therefore, results showed that most respondents indicated that their phones (99), routers (65), and modems (52) were enabled for internet connection using the 4G. Generally, more respondents indicated the 4G as their routinely used generation of RFR device. An inference from this report would be that mobile phones were the most important source of RFR within this population. This might further warrant the need to investigate further their specific attributes of use, noting that the use of mobile phones as compared to other computerised RFR-enabled devices encourages proximity to the user’s body and it was the flexibility of changing its position relative to the body quite easily and possibly quite frequently. This finding might support certain previous reports, especially those that identified mobile phones as a major source of RFR that is of health concern with respect to their deleterious effects on certain mental health attributes [29]. It is also worthy of note that certain people have been reported to be relatively hypersensitive to RFR exposure effects.

Overall, 81% of respondents indicated that they used their RFR-enabled devices every day. It is clear that in line with the global realities of technology diffusion and integration, almost every young person uses a device that could be connected to the internet [30]. The implication of this is that a significant proportion of the population and majority of the younger generation is exposed to RFR from their use(s) of technology. Narayanan et al. [21], for instance, had stated that the average adult uses a mobile phone for approximately 4-5 hours per day, as against the findings from this study that indicated 7-12 hours, and largely on daily basis. It is also possible that the changes in the educational systems and lifestyle as influenced by the COVID-19 pandemic has altered the patterns of technology use, such as by encouraging prolonged and more frequent use of the devices.

Data on perception and experiences with respect to RFR exposure through devices showed that RFR had effects on specific mental health and behavioural attributes especially causing sleep disturbances, fatigue and headache. This study also showed that many typical users of RFR-enabled devices had only average knowledge about RFR waves and their specific potential effects on mental health, thus raising concerns on the safety and safe use of devices. Based on the bivariate analysis, it could also be observed that urban dwellers were better informed on the nature and effects of RFR on their mental health, hence indicating the role of socioeconomic factors (Table 4). It is therefore strongly recommended that significant research investment is required in understanding the specific effects of RFR on mental health using diverse research methods and approaches, especially by aligning and combining experimental neuroscience and epidemiological methods. This has become very important as the world is increasingly embracing technology, many of which currently use RFR, and with a trend that predicts a monumental increase in RFR generation and exposure in the years to come with advancements in RER-enabled devices.

Limitation

The studied population largely had a range of 3G-4G internet service being provided with 4G being predominant; this study has therefore largely considered 4G RFR-enabled devices. Future research should also consider the 5G RFR-enabled devices, and possibly newer generations.

Furthermore, the setting of the current study was Southwestern Nigeria, noting that the level of infrastructural development and technology use could vary across the various regions, other regions could be studied as well.

Conclusion and recommendation

Data from the current total population survey showed that respondents' experiences and perception were that RFR had effects on certain mental health attributes, mainly, sleep disturbances, fatigue and headache. Also, the level of knowledge and awareness about RFR was at best average. This calls for concerns. It also calls for awareness programmes and proper education of the general populace. This points to an urgent need for awareness and education of the public on RFR-related mental health effects and the need for a culture of responsible deployment and use of RFR-enabled devices.

What is already known on this topic:

1. RFR exposure portrays health risks, and the RFR-health risks are associated with the dose and duration of exposure.

2. Most reports have only explored the effects of RFR exposure in relation to tumorigenesis, and interaction with genetic material has been a critical link to the cause of damage. 

3. Policies and recommendations have attempted to address RFR exposure and dose based on its suspected linkage to tumorigenesis

What this study adds:

1. Self-reported behavioural effects and changes that are associated with RFR exposure mainly included fatigue, sleep disturbances and headaches.

2. Populations exposed to RFR was high, with the most important source being mobile phones.

3. General population's knowledge about the nature and effects of RFR was just about average, hence, warrants awareness and further education of the populace.

## Conclusions

Data from the current total population survey showed that people perceived that RFR had effects on their mental health attributes, mainly, sleep disturbances, fatigue and headache. Also, the level of knowledge and awareness about RFR was at best average. This calls for concerns. It also calls for awareness programmes and proper education of the general populace. This points to an urgent need for awareness and education of the public on RFR-related mental health effects and the need for a culture of responsible deployment and use of RFR-enabled devices.
